# Supplementary material for: African-specific molecular taxonomy of prostate cancer
Source: Nature. 2022 Aug 31;609(7927):552–9. doi: 10.1038/s41586-022-05154-6 (PMC9477733; doi:10.1038/s41586-022-05154-6)
Supplement: Supplementary file 1 — Supplementary Information 1–11, including Supplementary Figs. 1–5, Supplementary Tables 13 and 14, and a guide to Supplementary Tables 1–12. [file 41586_2022_5154_MOESM1_ESM.pdf]

nature portfolio

https://doi.org/10.1038/s41586-022-05154-6

Supplementary information

African-specific molecular taxonomy of prostate cancer

In the format provided by the
authors and unedited

African-specific prostate cancer molecular taxonomy

Weerachai Jaratlerdsiri[1,2], Jue Jiang[1,2], Tingting Gong[1,2], Sean M. Patrick[3], Cali Willet[4], Tracy Chew[4], Ruth J. Lyons[2], Anne-Maree Haynes[2], Gabriela Pasqualim[5,6], Melanie Louw[7], James G. Kench[8], Raymond Campbell[9], Lisa G. Horvath[2,10], Eva K.F. Chan[2], David C. Wedge[11], Rosemarie Sadsad[4], Ilma Simoni Brum[5], Shingai B.A. Mutambirwa[12], Phillip D. Stricker[2,13], M.S. Riana Bornman[3], Vanessa M. Hayes[1,2,3,14*]

[1]Ancestry and Health Genomics Laboratory, Charles Perkins Centre, School of Medical Sciences, Faculty of Medicine and Health, University of Sydney, Camperdown, NSW, Australia; [2]Genomics and Epigenetic Theme, Garvan Institute of Medical Research, Darlinghurst, NSW, Australia; [3]School of Health Systems & Public Health, University of Pretoria, South Africa; [4]Sydney Informatics Hub, University of Sydney, Darlington, NSW, Australia; [5]Endocrine and Tumor Molecular Biology Laboratory (LABIMET), Instituto de Ciências Básicas da Saúde, Universidade Federal do Rio Grande do Sul, Brazil; [6]Laboratory of Genetics, Instituto de Ciências Biológicas, Universidade Federal do Rio Grande, Brazil; [7]National Health Laboratory Services, Johannesburg, South Africa; [8]Department of Tissue Pathology and Diagnostic Oncology, Royal Prince Alfred Hospital and Central Clinical School, University of Sydney, Sydney, NSW, Australia; [9]Kalafong Academic Hospital, Pretoria, South Africa; [10]Medical Oncology, Chris O'Brien Lifehouse, Royal Prince Alfred Hospital and Faculty of Medicine and Health, University of Sydney Camperdown, NSW, Australia; [11]Division of Cancer Sciences, University of Manchester, United Kingdom; [12]Department of Urology, Sefako Makgatho Health Science University, Dr George Mukhari Academic Hospital, Medunsa, South Africa; [13]Department of Urology, St. Vincent's Hospital, Darlinghurst, NSW, Australia; [14]Faculty of Health Sciences, University of Limpopo, Turfloop Campus, South Africa.

*e-mail: vanessa.hayes@sydney.edu.au

Supplementary Methods

Table of Contents

1. Patient cohort 4
2. Ethics 4
3. Whole-genome sequencing 4
4. Whole-genome sequencing analysis pipelines 5
4.1 Quality control 5
4.2 Variant discovery pipelines 5
4.2.1 Pipeline 1: Data pre-processing for variant discovery 7
4.2.2 Pipeline 2: Germline short variant discovery 9
4.2.3 Pipeline 3: Somatic short variant discovery 10
4.2.4 Pipeline 4: Structural variant discovery 10
4.3 Computing resources 11
4.4 Detailed computational resources and PBS job configurations 11
5. Population structure analysis 12
6. Inference of chromothripsis and chromoplexy 13
7. Mutational recurrence analysis 14
7.1 Driver mutations 14
7.2 Recurrent copy number alterations (CNAs) 14
7.3 Recurrent structural variation (SV) breakpoints 15
7.4 PCAWG somatic drivers 16
7.5 Tumours with no apparent drivers 16
8. Prostate cancer taxonomy 16
8.1 Integrative clustering analysis 16
8.2 Statistical significance of prostate cancer subtypes 17
8.3 Pathway and network analysis 18
8.4 Comparative cohorts 18
8.4.1 High-risk CPGEA 18
8.4.2 PCAWG 19

8.4.3 AACR GENIE 19
9. Mutational signature analysis 20
9.1 SBS, DBS and ID signatures 20
9.2 CN signatures..21
9.3 SV signatures 21
9.4 Statistical analysis of mutational signatures 22
10. Cancer evolution analysis 22
10.1 Clonal architecture analysis 22
10.2 Mutation timing 23
10.3 League model relative ordering 24
10.4 Reconstruction of prostate cancer timelines 24
11. References..26

1. Patient cohort

Our patient cohort was comprised of 183 patients from Australia (n=53), Brazil (n=7) and South Africa (n=123) who presented mostly with clinicopathologically confirmed prostate cancer. All except one Australian patient (PID 15178) treated with one-month-long Ozurdex therapy were treatment naïve at time of sampling. Three patients were unconfirmed for the cancer and confirmed for benign prostate hyperplasia (BPH). All men from the Southern African Prostate Cancer Study (SAPCS) were recruited at the time of diagnosis, and therefore tumour tissue was derived from biopsy core, while age and PSA levels were recorded at the time of diagnosis. Australian and Brazilian subjects were recruited at the time of radical prostatectomy. Their ages and PSA levels were also recorded at the same time. Additional selection criteria included: the availability of fresh-frozen tissue and matched blood, self-reported ethnicity, and country of origin, as well as the availability of clinical and pathological data (Supplementary Table 1).

2. Ethics

All samples were obtained with written informed consent, including consent to publication, as per study approval granted from the St. Vincent's Human Research Ethics Committee in Australia (HREC), SVH/12/231, the Grupo de Pesquisa e Pós-Graduação (GPPG) Scientific Committee and Research Ethical Commission (IRB) approval number 20160539 in Brazil or the University of Pretoria Faculty of Health Sciences Research Ethics Committee (with US Federal wide assurance FWA00002567 and IRB00002235 IORG0001762) approval number 43/2010 in South Africa. Samples were shipped to the Garvan Institute of Medical Research in accordance with institutional Material Transfer Agreements (MTAs), as well as additional Republic of South Africa Department of Health Export Permit (National Health Act 2003, J1/2/4/2 No 1/12). Whole-genome sequencing and analysis was performed in accordance with approval granted by St. Vincent's Hospital HREC SVH/15/227 and governance review authorisation granted for human research at the Garvan Institute of Medical Research GHRP1522.

3. Whole-genome sequencing

DNA was extracted from tissue and matched blood using either the DNeasy blood and tissue kit protocol (Qiagen, Maryland) or the Bionano Prep Frozen Human Blood and Animal Tissue DNA isolation protocols (Bionano Genomics, San Diego document #30246 and #30077 for high molecular weight DNA). All samples were processed through a single sequencing workflow at the Kinghorn Centre for Clinical Genomics at the Garvan Institute of Medical Research, with country-specific samples intermixed to avoid batch effects. The DNA underwent 2 x 150 bp sequencing on the Illumina NovaSeq instrument, with 21 patients sequenced using the Illumina HiSeq X Ten instrument (Supplementary Table 1).

4. Whole-genome sequencing analysis pipelines

Tracy Chew, Cali Willet, Jue Jiang, Tingting Gong, Weerachai Jaratlerdsiri, Eva K.F. Chan, Vanessa M. Hayes, Rosemarie Sadsad

The Sydney Informatics Hub (SIH), Core Research Facilities, University of Sydney developed the whole-genome sequencing analysis pipeline used in this study and optimised these pipelines for the University of Sydney's High Performance Computing cluster, Artemis, and Australia's National Computational Infrastructure (NCI), Gadi High Performance Computing facility.

4.1 Quality control

QC-tools can be used to quality check raw sequencing files. Supplementary Table 1 shows MultiQC reports of raw sequencing reads (FASTQ format) from Kinghorn Centre for Clinical Genomics (KCCG), Garvan Institute of Medical Research.

4.2 Variant discovery pipelines

Whole-genome sequencing data from 190 patients admitted to prostate cancer clinics (380 tumour and blood samples) were analysed at scale using four key pipelines (Supplementary Figures 1 and 2): *i*) data pre-processing for variant discovery, *ii*) germline short variant discovery, *iii*) somatic short variant discovery, and *vi*) structural variant discovery. The pipelines used either physical data chunking (Pipeline 1) or genomic interval chunking (Pipelines 1, 2, and 3) to divide the data for massively parallel processing. For jobs (Pipeline 4) where physical or interval chunking was not biologically valid, we

implemented a parallel-by-sample approach. Key algorithms that consumed the most compute resources will be discussed below.

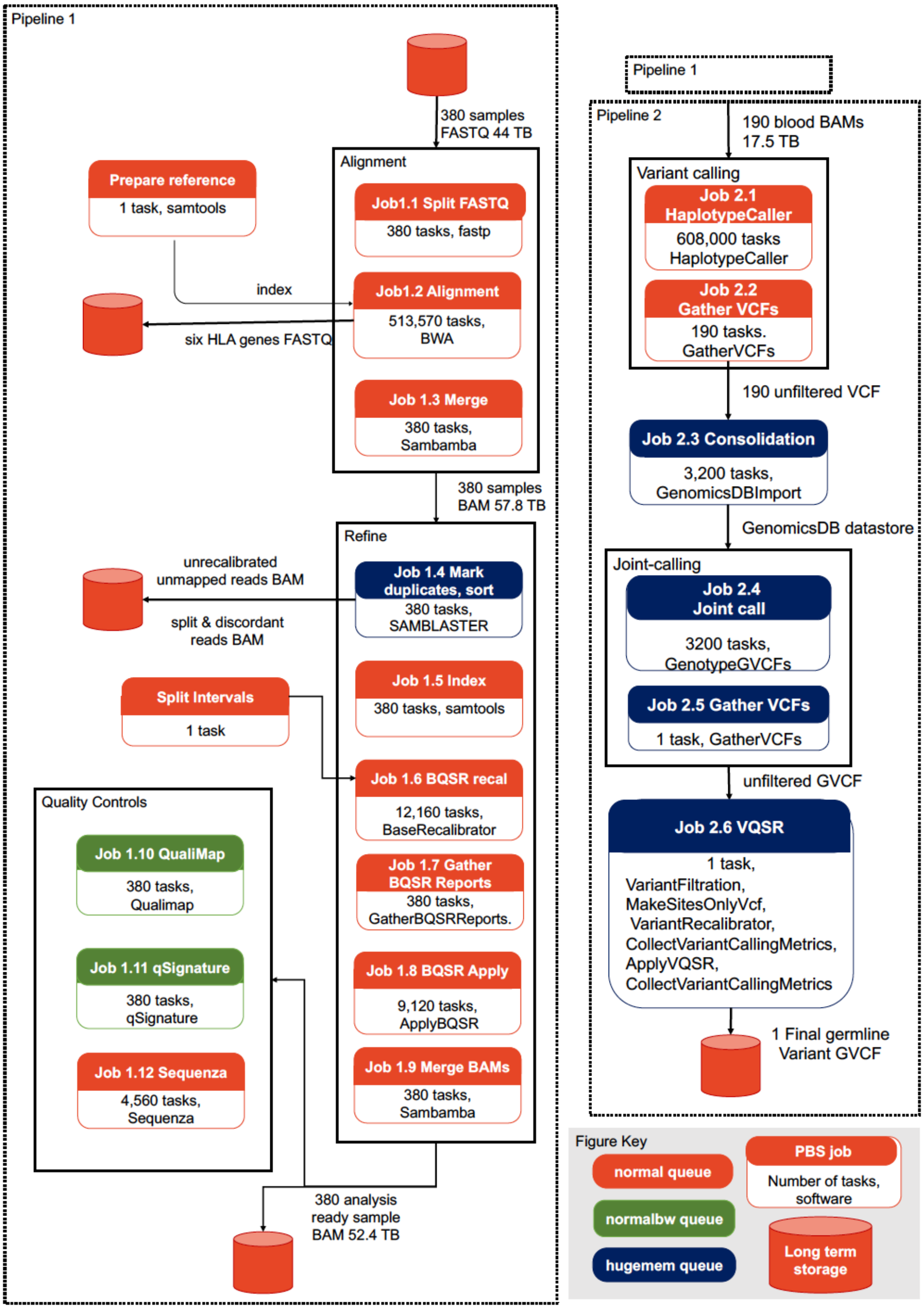

Supplementary Figure 1. Flowchart of Pipelines 1 and 2. For each PBS (Portable Batch System) job, a total number of tasks and which tool to be used can be found in the job box. Input and output files for each process are labelled with arrows.

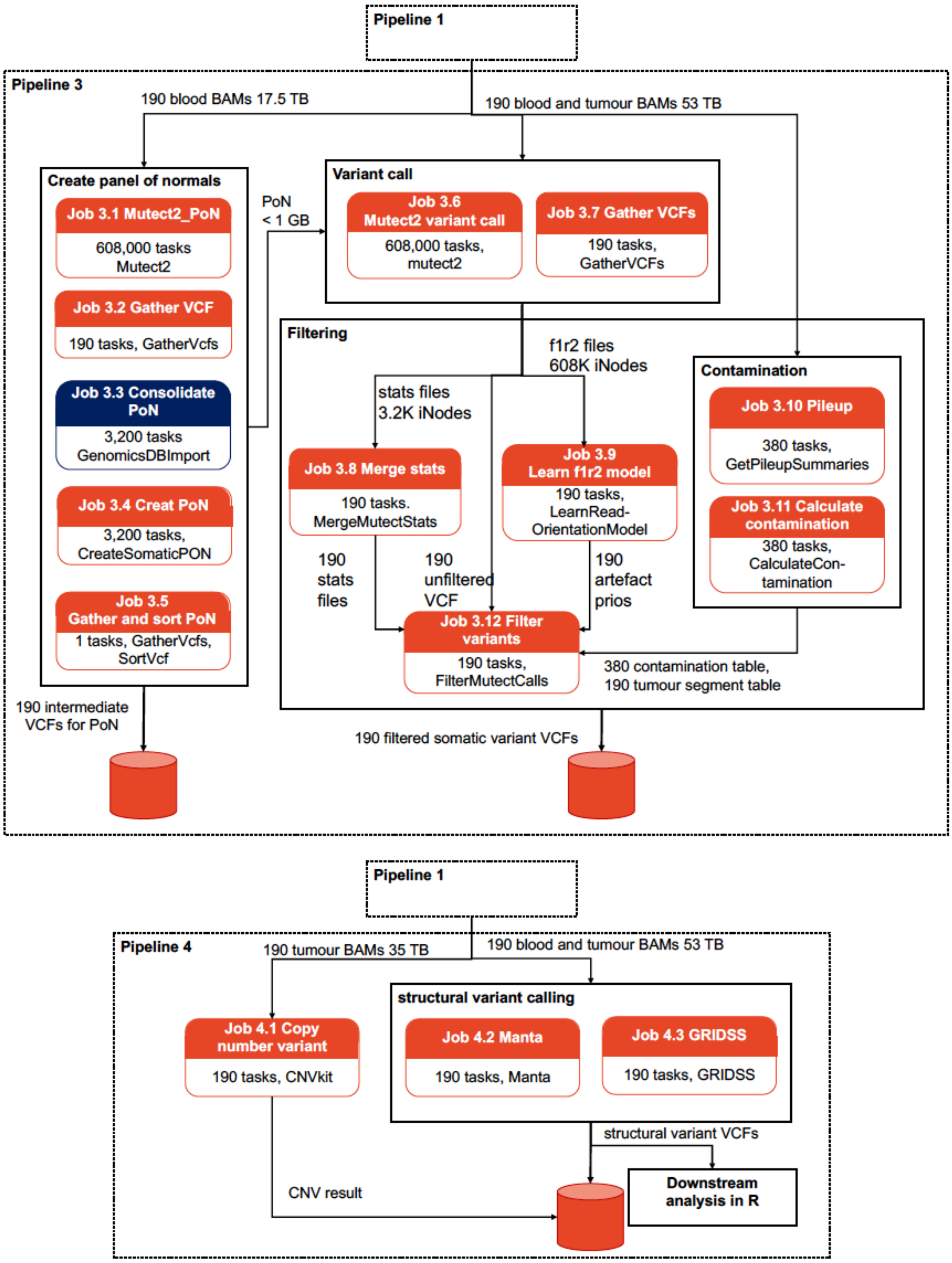

Supplementary Figure 2. Flowchart of Pipelines 3 and 4.

4.2.1 Pipeline 1: Data pre-processing for variant discovery

Data pre-processing for variant discovery is executed using the Fastq-to-BAM v2.0 pipeline[1]. Each stage of this pipeline is described in detail here. To prepare raw reads from the KCCG sequencing centre for this data pre-processing step, FASTQ files were separated by sequencing lane, using fastqsplit (https://github.com/supernifty/fastqsplit). Reads were adapter-trimmed and filtered using TrimGalore

v0.6.5 to remove low-quality bases (<Q15), short reads (<70bp), and missing read pairs. We also removed sequins, the synthetic DNA spike-in controls added during sequencing with Anaquin v3.9.0[2].

Each lane of filtered reads was aligned against human reference hg38 + alternate contigs using bwa v0.7.15[3]. BWAkit and BWA-mem functions were used concurrently to improve mapping quality scores for the primary human reference genome and a list of alternative haplotypes, therefore enabling variant calling. Each read pair was aligned as an independent entity, where we parallelised this job by first splitting the FASTQ files into smaller files of 500,000 reads (Job 1.1). These read data are homogenous in size, and each ran independently (Supplementary Figure 3). The scattered alignment tasks were merged with multi-threaded Sambamba v0.7.0 to produce one BAM per sample containing information about reads mapping to the human reference.

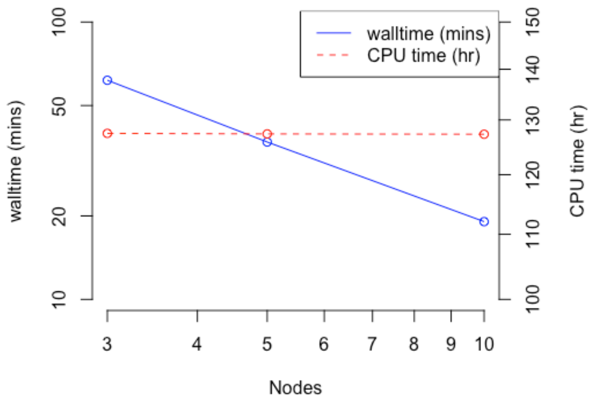

Supplementary Figure 3. Scalability of Job 1.2 alignment tested for a single blood sample

For analysis-ready BAM used in the following pipelines, duplicate reads (technical artefacts from the sequencing process) were marked using SAMBLASTER v0.1.24, and systematic error correction on the quality scores of DNA sequencing was performed using Base Quality Score Recalibration, GATK v4.1.2.0 (BQSR; Job 1.6). As per a scatter-gather method, recalibrated tables of each sample were generated on 3,367 contigs (3,366 natural hg38 contigs and one unmapped group). The final job merged

the scattered, recalibrated interval BAM files into an analysis-ready BAM file per sample with the Sambamba program.

For the last part of Pipeline 1, contaminated and mislabelled samples were estimated using qSignature v0.1 (Job 1.11). In this study, six tumour samples were removed for their comparison scores greater than 0.2, and two patients were duplicated based on the average Euclidean distances of 0.025-0.038 (qSignature distances) (Supplementary Table 10), consistent with the contamination table described below by GATK CalculateContamination (Job 3.11). Quality control of analysis-ready BAM files was explored using QualiMap v2.2.1 (Job 1.10; Supplementary Table 1). Sequenza v2.1.2 estimated the cellularity and ploidy of tumour samples (Job 1.12).

4.2.2 Pipeline 2: Germline short variant discovery

Germline short variant discovery is executed using the Germline-ShortV v1.0 pipeline[4]. Each stage of this pipeline is described in detail here. Germline short variants, including single nucleotide variants (SNV) and indels from 190 blood samples, were identified through the four following processes (Supplementary Figure 1): *i*) variant calling; *ii*) consolidation; *iii*) joint calling; and *vi*) variant quality score recalibration (VQSR).

The GATK HaplotypeCaller (Job 2.1) called germline variants by identifying nucleotide differences between the blood samples against 3,200 evenly sized intervals of the hg38 genome, using analysis-ready BAMs from Pipeline 1. Results were then merged into one variant call format (VCF) per sample with GatherVCFs (Job 2.2, 190 tasks). The unfiltered variant data of 190 blood samples were consolidated into a datastore format using the GATK GenomicsDBImport (Job 2.3), making the data more accessible and subsequently joint genotyped with GenotypeGVCFs (Job 2.4) using 3,200-interval chunking for efficient execution. After concatenating all the intervals, the joint genotyped VCF was filtered using the GATK Variant Quality Score Recalibration (VQSR; Job 2.6) algorithm, which trains a Gaussian mixture model with the profile of known variants. Variant quality score log-odds (VQSLOD), indicating the possibility of being a true positive versus a false positive, were assigned for each variant, with the sensitivity cut-off at 99.7. For a quick manual inspection, the quality of filtered variants was summarised using the GATK CollectVariantCallingMetrics.

4.2.3 Pipeline 3: Somatic short variant discovery

Somatic short variant discovery is executed using the Somatic-ShortV v1.0 pipeline[5]. Each stage of this pipeline is described in detail here. According to somatic short variant discovery (SNV and Indel) best practices by the BROAD Institute (GATK v4.1.2.0), the analysis-ready BAM files described in Section 4.2.1 were required for the following steps: *i*) creating the panel of normals (PoN), *ii*) conducting variant calling on the tumour samples, and *iii*) filtering away germline variants from the tumour variants using the PoN (Supplementary Figure 2).

To create the PoN for a control of germline variants and recurrent technical artefacts, the GATK Mutect2 (Job 3.1) was run in parallel for 3,200 intervals across blood samples. The results were consolidated into GenomicsDB datastore format to be processed as one large VCF file (Job 3.3). In the variant calling process, small somatic variants were identified across 3,200 intervals of the alignment of tumour-blood pairs using GATK Mutect2 that contrasted a variation of tumour tissue with the matched blood and PoN (Job 3.6).

The variants were filtered out for misalignment, strand and orientation bias, polymerase slippage, germline variation, and contamination. The GATK LearnReadOrientationModel read the f1r2 files generated from Mutect2 on interval to identify and filter out erroneous variants with higher frequency in one read pair orientation. The contamination was estimated with GATK CalculateContamination (Job 3.11), using the outputs of GATK GetPileupSummaries (Job 3.19), which summarised information of reads supporting known variants. The final step of filtering took account of all the information and evaluation from Job 3.8-3.11 using GATK FilterMutectCalls (Job 3.12).

4.2.4 Pipeline 4: Structural variant discovery

Somatic copy number alterations for each tumour were identified using CNVkit v0.9.6 (Job 4.1; Supplementary Figure 2), with an average bin size of 200 and 10,000 bp. Analysis-ready tumour BAM was analysed against a pool of blood references. The references were calculated for coverage from BAM read depths and pooled from 183 blood samples, excluding contaminated and duplicated samples and including previously published samples from South Africa[6,7].

Somatic structural variants (SV) were integrated results of two callers: Manta v1.6.0 and GRIDSS v2.8.3 (Job 4.2-4.3). The two callers have different underlying algorithms and consistently outperform other callers compared[8]. Manta calls used herein were defined as 'PASS' in the VCF output. We ran GRIDSS with default options on tumour and normal BAM files, and the gridss_somatic_filter.R script assigned supported calls with 'PASS'. High-confidence SVs in this study were defined as those passed by either Manta or GRIDSS filtering criteria and presented in unfiltered sets from both callers. We merged SV calls if their reported breakpoint positions overlapped within 500 bp unless otherwise noted. The annotation of merged SV types followed the conventional Manta definition.

Pipeline 4 was searched against the whole genome at once for genomic rearrangements observed across different chromosomes. As it was not biologically valid for scatter-gather parallelism, we performed multithreading options available in our pipeline tools and applied a parallel-by-sample approach.

4.3 Computing resources

Computation for the study was performed on three High Performance Computing (HPC) systems (Supplementary Table 13). All massively parallelised computation was performed using the National Computational Infrastructure (NCI) Gadi.

Supplementary Table 13 | HPC resources available in this study

	NCI Gadi		University of Sydney Artemis		Garvan Wolfpack
Type	Cluster	High memory	Cluster	High memory	Cluster
Manufacturer	Fujitsu	Fujitsu	Dell	Dell	Dell
Cores	155,000	2592	7588	192	5000
Processor Type	Intel Xeon Cascade Lake Platinum 3.2GHz	Intel Xeon Cascade Lake Platinum 2.9 GHz	Intel Xeon E5-2680 2.50 GHz	Intel Xeon E7-8860 2.20 GHz	Intel Xeon 2.5GHz /AMD Interlagos 2.6GHz
Cores per node	48	28/32	24/32/48	24/64	28/64
RAM per node	190GB	1TB/3TB	123GB	6TB	512GB
Compute storage	20PB Lustre		1PB Lustre		1PB PanFS
Job Scheduler	PBS Pro	PBS Pro	PBS Pro	PBS Pro	Sun Grid Engine

Note – GB, gigabyte; TB, terabyte

4.4 Detailed computational resources and PBS job configurations

Supplementary Table 14 | Detailed overview of computational resources and PBS job configurations for every job in Pipelines 1–4 (>1 KSU compute allocation). Usage for 190 patients has been extrapolated.

Jobs	Total tasks	PBS jobs	Total nodes	Average concurrent tasks /job	Average Walltime (hrs)	Total KSUs	Output size (TB)	Output iNode (K)
**Pipeline 1**								
FASTQ files	-	-	-	-	-	-	44	3
Job 1.1 Split fastq	2,621	15	144	188	1.8	15.5	38.6	1102
Job 1.2 Alignment	493,474	42	2624	461	0.3	160	57.4	5136
Job 1.3 Merge BAMs	409	20	164	19	0.8	20.1	57.8	0.4
Job 1.4 Mark duplicates, sort	386	13	305	30	2.9	88.2	33.1	2
Job 1.6 BQSR recal	12,192	8	112	516	0.6	11.8	<1	12.2
Job 1.8 BQSR apply	1,283,060	8	683	2357	1	75	72.1	27.3
Job 1.9 Merge BAMs	752	18	300	46	0.7	34	52.4	0.8
Job 1.10 QualiMap*	86	3	15	29	3.3	5.3	<1	1.5
Job 1.11 qSignature*	141	5	39	28	1	4.9	<1	0.8
**Pipeline 2**								
Job 2.1 HC	669,109	9	369	1,968	1.2	71.6	7.6	1216
Job 2.3 Consolidation	3,200	22	88	43	3.4	43	3.8	694.8
Job 2.4 Joint call	3,200	11	32	140	3.7	16.5	<1	6.4
**Pipeline 3**								
Job 3.1 Mutect2 PoN	608,048	5	381	3,658	0.9	40.7	<1	1216
Job 3.6 Mutect2 variant call	608,184	7	270	1,541	0.5	49.7	<1	2432
Job 3.9 Learn f1r2 model	220	5	9	40	1.6	2.4	<1	<1
**Pipeline 4**								
Job 4.1 CNV*	126	12	22	12	2.8	8.4	<1	<1
Job 4.2 Manta*	144	21	15	7	2	3.3	<1	0.6
Job 4.3 GRIDSS*	171	174	29	1	22	60.8	<1	3.5

Note – 1 KSU = 1,000 service units (SU of computation). Computational usage includes testing and re-run of failed tasks.

* We performed some jobs on both NCI Gadi and SIH Artemis. Here we only list computational resources used on NCI Gadi.

5. Population structure analysis

Weerachai Jaratlerdsiri, Jue Jiang, Tracy Chew, Cali Willet, Rosemarie Sadsad, Riana M.S. Bornman, Vanessa M. Hayes

To estimate genetic ancestry within our cohort of 183 patients from different populations excluding contaminated and duplicated samples and including previously published samples from South Africa[6,7], we added germline variant data from KhoeSan Genome Project (KSGP) and processed them all through the joint genotyping and VQSR described in Section 4.2.2. The KSGP consisted of 224 genomes

sequenced at high depth (~41X coverage) and acted as in-house reference populations within Africa for this analysis. A total of 407 genomes were processed to keep only biallelic SNVs within autosomes passed after recalibrated. Using PLINK v2.00[9], we filtered the variant data based on the variant's missing rate greater than 10%, minor allele frequency under 5% (singleton variants uninformative for population clustering), and *P*-values for Hardy-Weinberg Equilibrium (HWE) failed at 0.0001. The subsequent dataset of 7,472,833 remaining variants was used for data analysis.

Genetic ancestry was estimated using fastSTRUCTURE v1.0[10]. The program uses variational Bayesian inference to best approximate the marginal likelihood of a very large variant dataset. We analysed the dataset with randomly chosen initial seeds and a varying number of ancestral populations ranging from K=2 to K=9. The logistic prior model was preferred, providing higher marginal likelihood values than a simple model. Structure plots were visualised using Pophelper v2.2.7 in R[11]. The python script, chooseK.py predicted the number of ancestral populations that maximises the marginal likelihood of the fastSTRUCTURE data and the minimum number of populations with a cumulative ancestry contribution of at least 99.99%[10].

6. Inference of chromothripsis and chromoplexy

Tingting Gong, Jue Jiang, Weerachai Jaratlerdsiri, Eva K.F. Chan, Vanessa M. Hayes

Chromothripsis present in whole-genome data was detected using ShatterSeek v0.4[12]. The program integrated both somatic SV and copy number alteration callsets from our 183 tumours to detect better clusters of breakpoints from SVs that were interleaved or bridged the regions by their breakpoints instead of being nested. In this analysis, the somatic SVs were defined as high confidence by one of the callers described in Section 4.2.4. Merging the SVs between callers was considered if they had matching SV types and their reported breakpoint positions were within five bp of each other. As guided, large insertions and SVs within chromosome Y were excluded. Integer copy number within each tumour was derived using the default threshold (-1.1, -0.25, 0.2, 0.7) implemented in the CNVkit described above for 200 bp binning. Any adjacent copy number segments with the same state were merged. High-confidence chromothripsis regions were reported if selection criteria were met following the program's

instruction, with a significance cut-off at 0.20 for adjusted P-values (FDR). The Run_shatterseek.R script is provided for more details (https://github.com/tgong1/Code_HRPCa).

Chromoplexic rearrangements per tumour were tested using ChainFinder v1.0.1[13]. The same SV callset used for chromothripsis and segmentation data by CNVkit (Section 4.2.4) were converted from hg38 to hg19 coordinates for each tumour (see the ChainFinder_input_hg19.R script for more details; https://github.com/tgong1/Code_HRPCa). Program parameters of sequencing data were set for the segmented copy number and significance threshold at 0.05. Chromoplexic chains were plotted using the Circos software (http://circos.ca).

7. Mutational recurrence analysis

Weerachai Jaratlerdsiri, Jue Jiang, Tingting Gong, Vanessa M. Hayes

7.1 Driver mutations

Driver mutations of prostate cancer in protein-coding genes and noncoding regulatory elements were identified using ActiveDriverWGS v1.0.1[14]. The program performs statistical analyses of the number of small somatic mutations (SSM; SNVs and indels) within a given genomic element for being significantly more mutated than adjacent background genomic sequences (50 kbp window). The genomic elements analysed were retrieved from syn5259886, PCAWG[15], including coding sequences, promoters, 5'-UTRs, 3'-UTRs, enhancers, small RNAs, lncRNAs (promoter and exon regions), and miRNAs (pre-miRNA, mature and promoter). The elements are based on transcripts described in GENCODE v19 (gc19) and a set of additional noncoding RNA transcript annotations. To avoid leakage of signals from known cancer drivers, missense mutations were excluded when analysing the noncoding regions. The analysis discarded hypermutated samples (30 mutations/Mb). The MutsigCV v1.4.1 software for exome data also ran for the mutational significance of SSMs among genic regions with the following adjustable covariates: expression, replication time, and chromatin state[16]. Both programs provided rather identical results of coding drivers.

7.2 Recurrent copy number alterations (CNAs)

Focal and arm-level CNAs inferred from segmented copy number data of 183 prostate tumours (10-kb binning; Job 4.1) were examined using GISTIC v2.0.23[17]. The median number of segments used in this analysis was equal to 9,880. The following parameters were set: t_amp=0.1, t_del=0.1, qv_thresh=0.10, cap=1.5, join_segment_size=4, res=0.05, gene_collapse_method=extreme, broad_len_cutoff=0.7, and conf_level=0.99. All deletion and amplification lesions reported with FDR <0.10 were annotated for genic regions using Ensembl Release 99 gene annotations (https://www.ensembl.org).

7.3 Recurrent structural variation (SV) breakpoints

Recurrence analysis of SV breakpoints among 183 tumours was performed using fishHook v0.1[15,18], where Gamma-Poisson regression identifies statistical enrichment of somatic breakpoints in a given genomic interval after corrected by genomic covariates. A total of 48,234 breakpoints observed in our cohort (Section 4.2.4) had their positions converted to the GRCh37 build and counted within genic regions defined by GENCODE v19. We only considered one breakpoint per sample in a given interval. The eligible territory of the analysis excluded the 35-bp universal mask (um35-hs37d5.bed.gz) described by Li [19]; this mask contains regions of low mappability and low complexity. Our optimal regression model covered 2,159.6 Mb of the eligible territory, spanned 57,819 hypotheses and used four covariates (out of 10 tested), including replication timing, gene density, C content and mappability (100mer). Multiple hypothesis testing for the model was the false discovery rate (FDR) using the Benjamini–Hochberg method.

Any breakpoints of somatic interchromosomal rearrangements significantly abundant outside main chromosomes or within alternate contigs were verified using optical genome mapping (Bionano Genomics, https://bionanogenomics.com). The technology allows megabase-length genome images/maps to be reconstructed and rearranged genomes to be visualised without loss of integrity[20]. High molecular weight DNA of two patients, 12543 and UP2360, was processed following Crumbaker, et al. [21] for DNA labelling and imaging, except for the non-nicking enzyme DLE-1 (BNG, Part #20351) used in the BNG Saphyr system. *De novo* assembly of single molecules into consensus genome maps was performed using the Bionano Access 1.5.2 software with the aligner RefAligner 10330.10436rel[22]. SVs were identified relative to the human reference genome, hg38 + alternate contigs, whose genome maps were bioinformatically deduced based on predicted DLE-1 (CTTAAG) motif sites.

In addition, recurrent somatic juxtapositions of SV breakpoints in this study (2-dimensional connections between distinct genomic loci) were investigated using Ginseng[13] (https://github.com/walaj/ginseng). No significance was found.

7.4 PCAWG somatic drivers

Known driver genes in coding and noncoding regions published in PCAWG[15,23,24] were explored in our 183 tumours. Those specific to prostate cancer genes were also included[21,25-28]; 70 more driver genes were collected. Among those 1,730 drivers, 649 were found in our cohort across the 11 genomic elements described in Section 7.1. Moreover, significantly recurrent breakpoints and juxtapositions spanning within 311 genes reported in PCAWG were searched in this study[15]. Visualising the top 300 cancer genes significantly mutated in Sections 7.1–7.4 was carried out using maftools v2.2.10[29].

7.5 Tumours with no apparent drivers

All our 183 prostate tumours have recurrent alterations, regardless of mutational types (Extended Data Fig. 1c). This might be the result of our focus on African samples with high-risk prostate cancer. About 53 patients did not have PCAWG coding drivers observed (derived from hg19; Extended Data Fig. 2a), although three of them had the drivers if using hg38 annotation data (Supplementary Table 2). Nine patients in our cohort showed only recurrent CNAs, without any small somatic mutations and SV breakpoints detected.

8. Prostate cancer taxonomy

Weerachai Jaratlerdsiri, Vanessa M. Hayes

8.1 Integrative clustering analysis

For a prostate cancer taxonomy purpose, integrative clustering using iClusterPlus[26,30] was computed based on whole-genome information of 183 patients, including small somatic mutations, SV breakpoints and somatic copy number alterations. We considered binary features of significant and known driver genes based on SSM and SV data to indicate the presence or absence of a driver in each sample (Section 7) and generated cohort-wise segmentation data for CNA data (10-kb binning; Job 4.1). The SSM and SV data were normalised by gene; the CNA data chosen within non-redundant regions (epsilon=0.005;

rmSmallseg=TRUE) were set with an adaptive dimension reduction. All the data types were integrated into a single feature matrix for 183 patients. Bayesian information criteria (BIC) were selected for the best sparse model in our integrative analysis for molecular classification. Following the TCGA[26], molecular taxonomy comprising seven groups of oncogenic drivers was also compared in this cohort.

Individual consensus clustering of each whole-genome data across different tumours was performed using ConsensusClusterPlus v1.50.0[31] in R. It evaluated a maximum of 20 clusters, with 1,000 iterations of hierarchical clustering and 80% subsampling. Euclidean distance was used with Ward's method for hierarchical clustering. The SSM and SV normalised data described above were run, and segmented copy number data from tumours for the clustering were converted to a data matrix of overlapping chromosomal regions comparing all the possible sample pairs using CNTools v1.42.0 in R.

8.2 Statistical significance of prostate cancer subtypes

Statistical associations among diverse data types of recurrent alterations in this study (Sections 7.1–7.4) were evaluated against prostate cancer subtypes. Their *P*-values between subtypes and driver genes or elements were computed according to the nature of the data levels for each pair: categorical *versus* categorical (two-sided Fisher's Exact test) and categorical *versus* continuous (one-way ANOVA test). Suboptimal SSM and SV recurrent alterations were also tested if there were: *i*) observed values greater than expected; *ii*) uncorrected *P*-values <0.05; and *iii*) the number of affected patients greater than two. GISTIC results for all data (Section 7.2) in $\log_2$ copy number were associated with prostate cancer subtypes across 27,217 genes using a linear model. The *P*-value was adjusted for a false discovery rate (FDR) using the Benjamini-Hochberg correction (BH) for multiple-testing bias.

In addition, gene-centric integration of significantly mutated genes observed above at any test was collated across SSM, SV and CNA alterations and verified for its association with our prostate cancer subtypes. The integrated data applied GISTIC results of $\log_2$ copy number per gene and sample that were then adjusted to either -0.20 or +0.20 if additional driver genes and recurrent SV breakpoints were present in a sample. If uncertain, the adjustment also considered regular copy number changes per gene and/or prostate cancer subtype. The copy number adjustment of the integrated data at -0.10 or +0.10 was also tested for an association with prostate cancer subtypes for a comparative purpose. The adjusted $\log_2$ copy number was treated as a dependent variable for the ANOVA analysis. Note that the threshold

at either ±0.1 or ±0.2 was identical for the results of genes preferentially mutated in specific tumour subtypes, except for one fewer gene for the latter (Supplementary Figure 4).

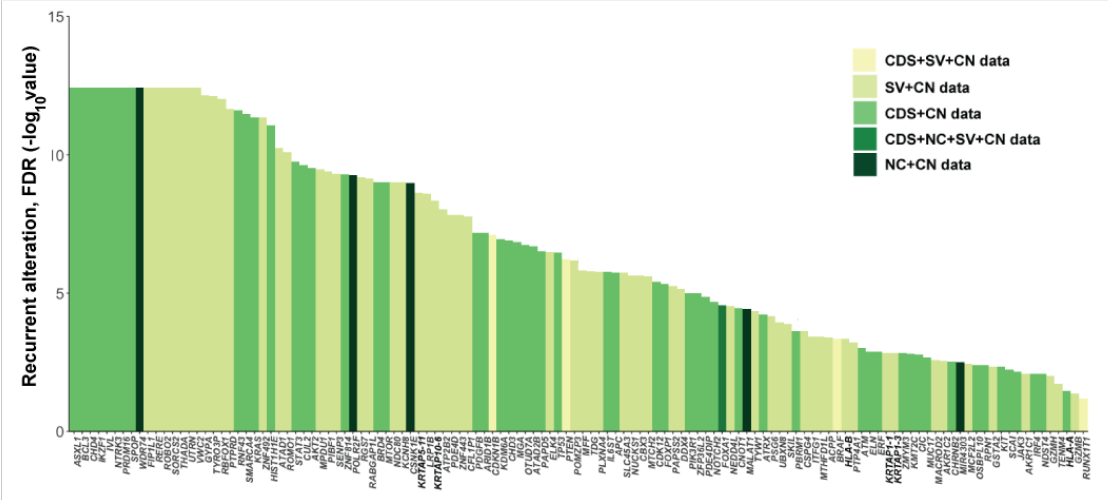

Supplementary Figure 4. A total of 124 genes associated with four prostate cancer subtypes. One-way ANOVA analyses were performed, and two-sided *P*-values were adjusted for a false discovery rate (FDR) using the Benjamini-Hochberg correction (BH) for multiple-testing bias. The Y-axis shows corrected *P*-values in $-\log_{10}$ values. CDS, coding driver data; NC, noncoding driver data; SV, significantly recurrent breakpoint data; and CN, gene-level copy number data.

8.3 Pathway and network analysis

The genes preferentially mutated in specific tumour subtypes mentioned above were used to discover enriched pathways using ActivePathways v1.0.2[32]. The program is an integrative method using a list of relevant genes across multiple datasets, including CNAs, SV, noncoding and coding drivers, and combining unadjusted *P*-values of the recurrent genes for all the datasets using Brown's extension[33]. The merged *P*-value was adjusted for false discovery using Holm's method. TCGA/ICGC cancer pathways were also searched among those genes, using maftools v2.6.05[29] and reported prostate cancer pathways by Armenia, et al[27]. Network-based visualisations of the enriched pathways were further carried out in the Cytoscape software[34], using the EnrichmentMap and AutoAnnotate against Gene Ontology (biological processes) and Reactome databases.

8.4 Comparative cohorts

8.4.1 High-risk CPGEA

To compare molecular subtypes within Asian prostate cancer, the Chinese Prostate Cancer Genome and

Epigenome Atlas (CPGEA, PRJCA001124), which is the largest and most comprehensive cancer genomics study conducted in China[35], was merged and processed with our integrative clustering analysis (Section 8.1). SSMs and somatic SV breakpoints from 93 high-risk prostate tumours were included and overlapped with our recurrent alterations (Section 7). Instead, GISTIC results for all data in $\log_2$ copy number (Section 7.2) from both high-risk CPGEA and this study were merged across 25,988 hg38-annotated genes and integrated with other data types for this analysis. Percent genome alteration (PGA) was calculated based on the total length of genes defined by the RefSeq database (UCSC GRCh38/hg38, Dec. 2013) and a cut-off at ±0.2. Gene-centric normalisation was performed in all the three datasets, and unsupervised hierarchical clustering was also run in each of them, using ConsensusClusterPlus v1.50.0[31] in R.

8.4.2 PCAWG

We leveraged the Pan-Cancer Analysis of Whole Genomes (PCAWG) to test tumour subtypes across different ethnic groups in other cancer types, using their SSM and SV consensus callsets and the GISTIC results for all data by gene in $\log_2$ copy number described above, as well as their sample demographic information[36]. We considered only cancers with patients of different primary ancestries at over 70% contribution (African, Asian and European): breast, liver, ovarian, and pancreatic cancers. With patients' black and gray lists excluded, this resulted in 101 breast, 254 liver, 55 ovarian, and 218 pancreatic cancer patients. Coding drivers in PCAWG included five mutation types (missense, splice site, nonsense, nonstop, and start codon), four deletion types (in-frame, frame-shift, stop codon, and start codon), and three insertion types (in-frame, frame-shift, and stop codon insertions). PGA was calculated based on the total length of 23,956 genes defined by the RefSeq database (UCSC GRCh37/hg19, Feb. 2009) and a cut-off at ±0.2. Separate and integrative hierarchical clustering analyses of each dataset were performed as mentioned in Section 8.4.1.

8.4.3 AACR GENIE

The AACR Project Genomics, Evidence, Neoplasia, Information, Exchange (GENIE) consists of 865 patients with primary prostate cancers (FFPE) and accompanying clinical data, including their primary race of Asian, Black or White (n=20, 56 and 789, respectively). All the patients have the following three

types of cancer genomic data available for exonic regions (Reference genome hg19): *i*) CDS mutations (183 genes); *ii*) gene fusions (183 genes); and *iii*) gene-level copy number alterations by GISTIC (176 genes). The patients are from two American cohorts, DFCI and MSK. The AACR GENIE data (version 11.0-public) were retrieved via Synapse Platform (https://synapse.org/genie)[37,38]. For comparisons, the same gene panels were extracted from CPGEA and our cohort and all the three cohorts proceeded with integrative clustering analysis (Section 8.1). Visualisation of prostate cancer subtypes is illustrated in Supplementary Figure 5, with GMS-A, -B, -C and -E present simultaneously with the proportion of altered genes across the panel examined per patient for the three mutational types.

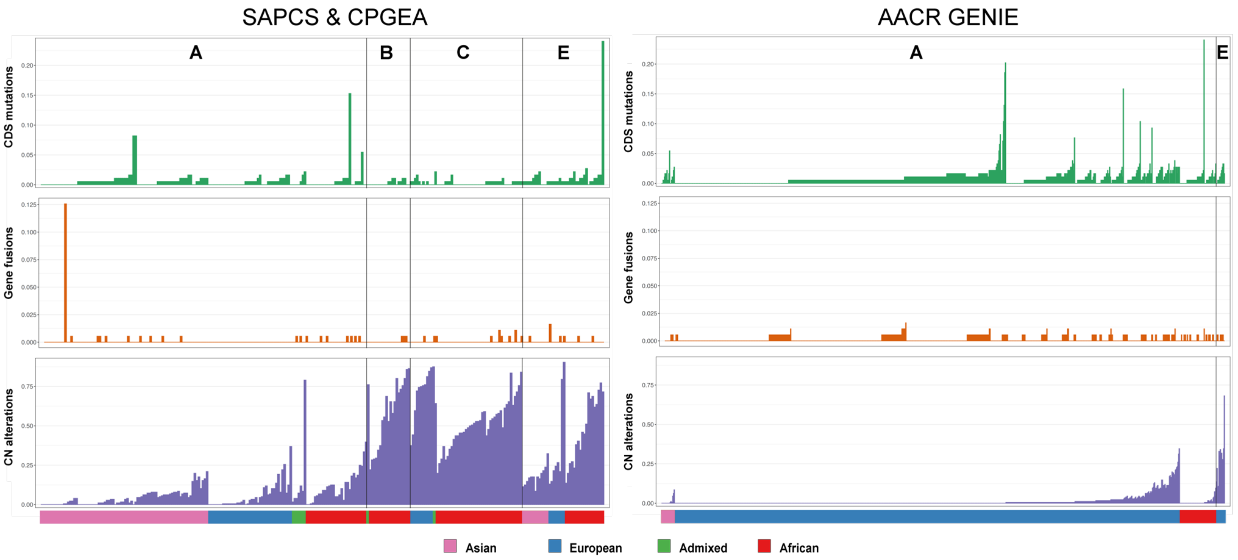

Supplementary Figure 5. Integrative clustering analysis of AACR GENIE patients with primary prostate tumours reveals two distinct molecular subtypes. The concurrent analysis with CPGEA and our cohort shows four subtypes, with GMS-D missing. The AACR GENIE includes African Americans identified as Black. Note that a level of genetic admixture among AACR GENIE samples, especially African Americans, is unknown.

9. Mutational signature analysis

Weerachai Jaratlerdsiri, David C. Wedge, Vanessa M. Hayes

9.1 SBS, DBS and ID signatures

96 SBS (single base substitution), 78 DBS (doublet base substitution) and 83 ID (small indel) classes of our high-confidence SSM dataset from 183 patients (Section 4.2.3) were analysed for a catalogue of

somatic mutational signatures using SigProfiler[39,40]. The program implements nonnegative matrix factorisation (NMF) detecting signature profiles and contributions of each signature to each tumour. The hierarchical *de novo* extraction of mutational signatures and their sequence context of the somatic mutations reported was also used for this analysis, in addition to the global COSMIC signatures (Catalogue of Somatic Mutations in Cancer v3.2, Mar. 2021). The following parameters were set: maximum_signatures=15, NMF_replicates=500, NMF_init=random, matrix_normalization=gmm, resample=TRUE, min_NMF_iterations=10000, max_NMF_iterations=1000000, clustering_distance=cosine, stability=0.8, opportunity_genome=GRCh38 (main chromosomes).

9.2 CN signatures

Copy number (CN) signatures and their mutational processes among 183 prostate tumours were identified using SigProfilerMatrixGenerator module in SigProfiler[39,40]. The module created a feature matrix of 45 CN features across all the tumours, given that multi-sample segmented data computed above by Sequenza (Job 1.12) were provided. The NMF package in R was computed and deconvoluted the patient-by-component sum-of-posteriors matrix into a patient-by-signature matrix and a signature-by-component matrix[41]. The NMF procedure performed 50 runs of random initialisations for a variety of ranks (1-15), using the Kullback-Leibler distance and Brunet algorithm. The final factorisation using the estimated rank and 200 runs defined the number of clusters to approximate the target matrix. The optimal factorisation rank considered a point where the cophenetic correlation coefficient started decreasing, and the residual sum of squares (RSS) curve presented an inflection point. Randomised data were also generated to confirm the lowest approximation error across multiple runs.

9.3 SV signatures

The patterns and signatures of structural variants across 183 tumours were studied according to the PCAWG's working classification and annotation scheme for genomic rearrangements[42]. First, exact breakpoint coordinates of somatic SVs were generated using two SV callers (Section 4.2.4). Second, rearrangement breakpoint and copy number data were merged to investigate a structural and mechanistic association between copy number segment and rearrangement. Adjusted $\log_2$-ratio of the segmented data for each tumour was computed using the CNVkit calling pipeline of integer copy number (10,000-bp binning; Section 4.2.4), with sample-specific ploidy and purity estimates obtained from Sequenza

(Job 1.12). Third, clustering SVs into clusters and their footprints was processed using ClusterSV (https://github.com/cancerit/ClusterSV/). The SV cluster shows several breakpoints occurring close together in time or genomic space, while the footprint is a genomic interval assumed to have undergone a complex rearrangement event. Fourth, redundant segment-bypassing SVs were removed if the total length of all the bypassed segments was less than SV insert size. Lastly, we refined those clusters and footprints and inspected them for their rearrangement patterns and categories. Considering WA scores per 1-kb loci from normal prostate epithelial cells (PrEC), replication timing for SVs within autosomes and chromosome X was defined as early (WA >75), mid (WA=2–75) and late categories (WA <20)[43]. Major fragile sites defined by PCAWG (n = 18) were also compared with our SV clusters[42]. We followed the steps of heuristic refinement of how the boundary and width of the footprints should be determined as described by Li, et al[42].

As per the above-mentioned classification, a feature matrix of counts per patient (across 183 patients) of SV clusters falling into different features of SVs split by size and/or replication timing was created and used for the NMF analysis described above to estimate an optimal factorisation rank and, therefore, the number of SV signatures in this cohort.

9.4 Statistical analysis of mutational signatures

The most enriched signatures for each mutational class (SBS, DBS, ID, CN and SV) within each patient were identified and associated with our PCa subtypes using linear regression with one-way ANOVA. Fisher's exact test was also performed for the association between signature and categorical variable. Both *de novo* and global COSMIC signatures were tested for SBS, DBS and ID classes. In addition, Spearman correlations of mutational signatures detected in this study were analysed with each of our clinical data and 32 significantly mutated genes observed in pan-cancer and prostate cancer studies (integrated data in Section 8.2). For multiple hypothesis testing, *P*-values were corrected with the FDR method.

10. Cancer evolution analysis

Weerachai Jaratlerdsiri, David C. Wedge, Vanessa M. Hayes

10.1 Clonal architecture analysis

Reconstructing clonal architecture and frequency from a tumour-normal pair was conducted using the TitanCNA snakemake workflow v1.17.1[44] and PhyloWGS[45]. The TitanCNA inferred copy number architectures in clonal cell populations from our 183 tumours. To improve accuracy, the alignment data of 183 blood samples (Section 4.2.1) were used as the PoN, with a 10-kb window size. The optimal clonal cluster per tumour was selected based on the S_Dbw validity index and manual inspection of the results. The PhyloWGS program defines multiple subpopulations of cancerous cells based on variant allele frequency (VAF) of somatic mutations corrected by copy number frequency. The program required the optimal CNAs adjusted by purity (Sequenza; Job 1.12) and filtered SSMs described in Section 4.2.3; we also removed any CNAs generated, with the total read depth of zero. This analysis also included normal copy number regions, so mutations spanning those regions could be merged and analysed using default settings with four MCMC chains. A best tree of clonal architecture and frequency was chosen with the lowest normalised log-likelihood (nlgLH), but the highest likelihood (LLH).

10.2 Mutation timing

To improve a cancer timeline estimated above by variant allele frequency (Section 10.1), physical alterations or whole-genome duplications (WGD) and the most recent common ancestor (MRCA) of all cancer cells in a tumour sample were marked and facilitated defining a major stage of tumour development on the accumulation of somatic mutations and CNAs, using MutationTimeR[46] in R. The program follows a beta-binomial distribution and considers discrete values of local copy number and subclonal composition. Filtered SSM calls (Section 4.2.3) were required with information including a tumour's reference and alternate allele counts; CNA data required were identical to ones used by PhyloWGS. Cellular prevalence and the number of SSMs per clone computed above in Section 10.1 (Supplementary Table 11) was provided for each male patient. Clonal frequency and tumour purity parameters were interchangeable in this analysis. In this study, the timing algorithm involving whole-genome duplication (bi-allelic gains) was mainly considered following Gerstung, et al[46]. Confidence intervals ($t_{lo} - t_{up}$) for timing estimates were calculated with 200 bootstraps.

According to Gerstung, et al[46], the rate of mutation acquisition prior to a patient's age at the time of the study was calculated based on clonal and subclonal branch length of mutation burden (6 Gb for a diploid genome) derived from MutationTimeR results (copy number frequency and subclonal posterior

probability). Per-sample CpG-to-TpG mutations from chromosomes 1-X (CpG>TpG; CGN or NCG) were counted for the analysis, as the mutations caused by spontaneous deamination of 5-methyl-cytosine to thymine at CpG dinucleotides have been proposed as a molecular clock. We then computed the median mutation rate for each prostate cancer subtype by summing all the scaled branch length and using a patient's age as a denominator.

10.3 League model relative ordering

League model relative ordering by aggregating the order of driver genes and CNAs across samples to define a probabilistic ranking of the drivers was performed using PhylogicNDT (https://github.com/broadinstitute/PhylogicNDT). Due to the different data types the program required at the time of analysis, we skipped the Clustering and SinglePatientTiming modules in the program and created a single aggregated table described for the LeagueModel module. Orderings of each pair of driver genes and CNAs (early, late, unspecified clones and subclones) were derived from the timing of each driver mutation and the timing status of clonal and subclonal copy number segments retrieved from MutationTimeR. The table of those orderings was aggregated across all samples with the drivers available (n=132/183). The probability of the first event in a pair occurring before the second one would be either '0' or '1'; otherwise, both events were '0' if unknown was '1'. Sports statistics in the League model were employed to calculate the overall ranking of driver events, with the following parameters: n_perms=1000, n_seasons=1000, percent_subset=0.90, and num_games_against_each_opponent=2.

10.4 Reconstruction of prostate cancer timelines

Results of the MutationTimeR described in Section 10.2, which gathered per-sample information of both VAF of somatic mutations and marked copy number gains and classified them into four stages (early clonal, unspecified clonal, late clonal and subclonal), were combined across samples in the same tumour subtype to reconstruct a timeline of cancer evolution. We included only driver mutations identified in this study (Sections 7.1 and 7.4) and recurrent copy number overlapped with GISTIC results (Section 7.2), genes preferentially mutated in specific tumour subtypes (Section 8.2) and/or relevant copy number altered within the study by Wedge, et al[28]. The copy number was overlapped for both alteration size (>50%) and type. The driver genes and copy number reported were present within at least two tumours, with the same timing annotation. Somatic mutations among the four timing periods

of the MutationTimeR in our 183 tumours were then profiled for their mutational signatures and compositions using SigProfiler (Section 9.1).

Mutually exclusive (OD <0.5) or co-occurring drivers (OD >2) were detected cohort-wise using somaticInteractions function in maftools v2.2.10, which performs pair-wise Fisher's exact test for pair-wise significance[29]. The recurrent copy number by GISTIC (FDR <0.10; Section 7.2) locally overlapped with copy number changes through time defined by Wedge, et al[28] were analysed together with the driver genes described above (Sections 7.1 and 7.4) across 183 patients, as well as consensus driver genes of additional 257 prostate cancer patients from PCAWG[36]

Each cancer timeline begins at the fertilised egg and spans up to the median age of the patients within each subtype[46]. WGD and the MRCA act as anchor points to separate between early and late clonal periods and between clonal and subclonal periods, respectively. A variable/constant time period includes events that are ranked before the WGD event and also begins shortly after another break in the timeline. The late period does have a definite start, as this includes events that are ranked after WGD, when it occurs. Specific driver genes or recurrent copy number can be placed within these time frames. COSMIC signatures reported are shown on the timeline if they fluctuate over time or contribute a substantial fraction of somatic mutations (at least 10% per timing period). The signature is annotated during the epoch of its greatest intensity.

11. References

1 Fastq-to-BAM v. 2.0 (https://doi.org/10.48546/workflowhub.workflow.146.1, 2021).

2 Blackburn, J. *et al.* Use of synthetic DNA spike-in controls (sequins) for human genome sequencing. *Nat Protoc* **14**, 2119-2151 (2019).

3 Li, H. & Durbin, R. Fast and accurate short read alignment with Burrows-Wheeler Transform. *Bioinformatics* **25**, 1754-1760 (2009).

4 Germline-ShortV v. 1.0 (https://doi.org/10.48546/workflowhub.workflow.143.1 2021).

5 Somatic-ShortV v. 1.0 (https://doi.org/10.48546/workflowhub.workflow.148.1 2021).

6 Jaratlerdsiri, W. *et al.* Whole Genome Sequencing Reveals Elevated Tumor Mutational Burden and Initiating Driver Mutations in African Men with Treatment-Naïve, High-Risk Prostate Cancer. *Can Res* **78**, 6736-6746 (2018).

7 Jaratlerdsiri, W. *et al.* Next generation mapping reveals novel large genomic rearrangements in prostate cancer. *Oncotarget* **8**, 23588-23602 (2017).

8 Gong, T., Hayes, V. M. & Chan, E. K. F. Shiny-SoSV: A web-based performance calculator for somatic structural variant detection. *PLoS One* **15**, e0238108 (2020).

9 Chang, C. C. *et al.* Second-generation PLINK: rising to the challenge of larger and richer datasets. *Gigascience* **4**, 7 (2015).

10 Raj, A., Stephens, M. & Pritchard, J. K. fastSTRUCTURE: variational inference of population structure in large SNP data sets. *Genetics* **197**, 573-589 (2014).

11 RStudio-Team. *RStudio: Integrated Development for R.* (RStudio, Inc., 2015).

12 Cortés-Ciriano, I. & Lee JJ, X. R., Jain D, Jung YL, Yang L, Gordenin D, Klimczak LJ, Zhang CZ, Pellman DS; PCAWG Structural Variation Working Group, Park PJ; PCAWG Consortium. Comprehensive analysis of chromothripsis in 2,658 human cancers using whole-genome sequencing. *Nat Genet* **52**, 331–341 (2020).

13 Baca, S. C. *et al.* Punctuated evolution of prostate cancer genomes. *Cell* **153**, 666-677 (2013).

14 Zhu, H. *et al.* Candidate Cancer Driver Mutations in Distal Regulatory Elements and Long-Range Chromatin Interaction Networks. *Mol Cell* **77**, 1307-1321.e1310 (2020).

15 Rheinbay, E. *et al.* Analyses of non-coding somatic drivers in 2,658 cancer whole genomes. *Nature* **578**, 102-111 (2020).

16 Lawrence, M. S. *et al.* Mutational heterogeneity in cancer and the search for new cancer-associated genes. *Nature* **499**, 214-218 (2013).

17 Mermel, C. H. *et al.* GISTIC2.0 facilitates sensitive and confident localization of the targets of focal somatic copy-number alteration in human cancers. *Genome Biol* **12**, R41 (2011).

18 Imielinski, M., Guo, G. & Meyerson, M. Insertions and Deletions Target Lineage-Defining Genes in Human Cancers. *Cell* **168**, 460-472.e414 (2017).

19 Li, H. Toward better understanding of artifacts in variant calling from high-coverage samples. *Bioinformatics* **30**, 2843-2851 (2014).

20 Chan, E. K. F. *et al.* Optical mapping reveals a higher level of genomic architecture of chained fusions in cancer. *Genome Res* **28**, 726-738 (2018).

21 Crumbaker, M. *et al.* The Impact of Whole Genome Data on Therapeutic Decision-Making in Metastatic Prostate Cancer: A Retrospective Analysis. *Cancers (Basel)* **12**, E1178 (2020).

22 Lam, E. T. *et al.* Genome mapping on nanochannel arrays for structural variation analysis and sequence assembly. *Nat Biotechnol* **30**, 771-776, doi:10.1038/nbt.2303 (2012).

23 Martincorena, I. *et al.* Universal Patterns of Selection in Cancer and Somatic Tissues. *Cell* **171**, 1029-1041.e1021 (2017).

24 Lawrence, M. S. *et al.* Discovery and saturation analysis of cancer genes across 21 tumour types. *Nature* **505**, 495-501 (2014).

25 Robinson, D. *et al.* Integrative clinical genomics of advanced prostate cancer. *Cell* **161**, 1215-1228 (2015).

26 The-Cancer-Genome-Atlas-Network. The molecular taxonomy of primary prostate cancer. *Cell* **163**, 1011-1025 (2015).

27 Armenia, J. *et al.* The long tail of oncogenic drivers in prostate cancer. *Nat Genet* **50**, 645-651 (2018).

28 Wedge, D. C. *et al.* Sequencing of prostate cancers identifies new cancer genes, routes of progression and drug targets. *Nat Genet* **50**, 682-692 (2018).

29 Mayakonda, A., Lin, D. C., Assenov, Y., Plass, C. & Koeffler, H. P. Maftools: efficient and comprehensive analysis of somatic variants in cancer. *Genome Res* **28**, 1747-1756 (2018).

30 Mo, Q. *et al.* Pattern discovery and cancer gene identification in integrated cancer genomic data. *Proc Natl Acad Sci U S A* **110**, 4245-4250 (2013).

31 Wilkerson, M. D. & DN., H. ConsensusClusterPlus: a class discovery tool with confidence assessments and item tracking. *Bioinformatics* **26**, 1572-1573 (2010).

32 Paczkowska, M. *et al.* Integrative pathway enrichment analysis of multivariate omics data. *Nat Commun* **11**, 735 (2020).

33 Brown, M. B. A method for combining non-independent, one-sided tests of significance. *Biometrics* **31**, 987–992 (1975).

34 Reimand, J. *et al.* Pathway enrichment analysis and visualization of omics data using g:Profiler, GSEA, Cytoscape and EnrichmentMap. *Nat Protoc* **14**, 482-517 (2019).

35 Li, J. *et al.* A genomic and epigenomic atlas of prostate cancer in Asian populations. *Nature* **580**, 93-99 (2020).

36 ICGC/TCGA-Pan-Cancer-Analysis-of-Whole-Genomes-Consortium. Pan-cancer analysis of whole genomes. *Nature* **578**, 82-93 (2020).

37 Mahal, B. A. *et al.* Racial Differences in Genomic Profiling of Prostate Cancer. *N Engl J Med* **383**, 1083-1085 (2020).

38 AACR-Project-GENIE-Consortium. AACR Project GENIE: powering precision medicine through an international consortium. *Cancer Discov* **7**, 818-831 (2017).

39 Nik-Zainal, S. *et al.* Landscape of somatic mutations in 560 breast cancer whole-genome sequences. *Nature* **534**, 47-54 (2016).

40 Alexandrov, L. B. *et al.* The repertoire of mutational signatures in human cancer. *Nature* **578**, 94-101 (2020).

41 Gaujoux, R. & Seoighe, C. A flexible R package for nonnegative matrix factorization. *BMC Bioinformatics* **11**, 367 (2010).

42 Li, Y. *et al.* Patterns of somatic structural variation in human cancer genomes. *Nature* **578**, 112-121 (2020).

43 Du, Q. *et al.* Replication timing and epigenome remodelling are associated with the nature of chromosomal rearrangements in cancer. *Nat Commun* **10**, 416 (2019).

44 Ha, G. *et al.* TITAN: inference of copy number architectures in clonal cell populations from tumor whole-genome sequence data. *Genome Res* **24**, 1881-1893 (2014).

45 Deshwar, A. G. *et al.* PhyloWGS: reconstructing subclonal composition and evolution from whole-genome sequencing of tumors. *Genome Biol* **16**, 35 (2015).

46 Gerstung, M. *et al.* The evolutionary history of 2,658 cancers. *Nature* **578**, 122-128 (2020).

SUPPLEMENTARY TABLE GUIDE

Supplementary Table 1 | Clinical cohort characteristics and sequencing quality

Supplementary Table 2 | Driver information by patient

Supplementary Table 3 | GISTIC2 results of all genomic lesions under 99% confidence level

Supplementary Table 4 | List of significantly recurrent SV breakpoints at FDR lower than 0.10

The effect size and two-sided *P*-values were computed using generalised linear modelling of somatic breakpoints in each genomic interval. The FDR was calculated using the Benjamini–Hochberg method.

Supplementary Table 5 | TCGA prostate cancer taxonomy identified in this study

Patient by driver mutation and patient by driver structural variation summary matrices are provided.

Supplementary Table 6 | Integrative iCluster analysis of 183 prostate tumours

Supplementary Table 7 | List of 124 preferentially mutated genes within four tumour subtypes

Supplementary Table 8 | Pathway enrichment analysis of 124 preferentially mutated genes

The g:Profiler program performs a gene-set enrichment analysis using a hypergeometric test (Fisher's Exact test), with the Benjamini-Hochberg false discovery rate procedure applied for one-sided *P*-value adjustment.

Supplementary Table 9 | Total mutational signature profiles across 183 tumours

The table shows data matrices of SBS feature by patient, DBS feature by patient, ID feature by patient, CN feature by patient, and SV feature by patient. For each type, the most enriched signature per patient is recorded to compare with GMS subtypes.

Supplementary Table 10 | Cross-individual contamination level

Supplementary Table 11 | Cancer evolution analysis of prostate cancer

Clonal architecture by PhyloWGS and timing of gains and drivers by MutationTimeR is provided per tumour

Supplementary Table 12 | Summary of major differences between African and European prostate cancer genomes

Supplementary Table 13 | HPC resources available in this study

Supplementary Table 14 | Detailed overview of computational resources and PBS job configurations for every job in Pipelines 1–4 (>1 KSU compute allocation). Usage for 190 patients has been extrapolated.
