# Supplementary material for: African-specific molecular taxonomy of prostate cancer
Source: Nature. 2022 Aug 31;609(7927):552–9. doi: 10.1038/s41586-022-05154-6 (PMC9477733; doi:10.1038/s41586-022-05154-6)
Supplement: Supplementary file 2 — Reporting Summary [file 41586_2022_5154_MOESM2_ESM.pdf]

nature portfolio

Corresponding author(s): Vanessa M. Hayes

Last updated by author(s): Nov 28, 2021

Reporting Summary

Nature Portfolio wishes to improve the reproducibility of the work that we publish. This form provides structure for consistency and transparency in reporting. For further information on Nature Portfolio policies, see our Editorial Policies and the Editorial Policy Checklist.

Statistics

For all statistical analyses, confirm that the following items are present in the figure legend, table legend, main text, or Methods section.

n/a	Confirmed	
☐	☒	The exact sample size (*n*) for each experimental group/condition, given as a discrete number and unit of measurement
☐	☒	A statement on whether measurements were taken from distinct samples or whether the same sample was measured repeatedly
☐	☒	The statistical test(s) used AND whether they are one- or two-sided<br>*Only common tests should be described solely by name; describe more complex techniques in the Methods section.*
☐	☒	A description of all covariates tested
☐	☒	A description of any assumptions or corrections, such as tests of normality and adjustment for multiple comparisons
☐	☒	A full description of the statistical parameters including central tendency (e.g. means) or other basic estimates (e.g. regression coefficient) AND variation (e.g. standard deviation) or associated estimates of uncertainty (e.g. confidence intervals)
☐	☒	For null hypothesis testing, the test statistic (e.g. *F*, *t*, *r*) with confidence intervals, effect sizes, degrees of freedom and *P* value noted<br>*Give P values as exact values whenever suitable.*
☒	☐	For Bayesian analysis, information on the choice of priors and Markov chain Monte Carlo settings
☐	☒	For hierarchical and complex designs, identification of the appropriate level for tests and full reporting of outcomes
☐	☒	Estimates of effect sizes (e.g. Cohen's *d*, Pearson's *r*), indicating how they were calculated

Our web collection on statistics for biologists contains articles on many of the points above.

Software and code

Policy information about availability of computer code

Data collection	Data and metadata were collected from International Cancer Genome Consortium (ICGC), using ICGC Data Portal search (https://dcc.icgc.org/). Data repositories specific to CPGEA were also used for data collection (http://www.cpgea.com).
Data analysis	The core computational pipelines used in this study for read alignment, quality control and variant calling are available to the public at https://github.com/Sydney-Informatics-Hub/Bioinformatics. Analysis code for chromothripsis and chromoplexy is available through another GitHub page, https://github.com/tgong1/Code_HRPCa. Individual software components are as follows: fastp v.0.20.0; bwa v0.7.15; bwakit v. 0.7.17; SAMbamba v.0.7.1; SAMblaster v. 0.1.24; SAMtools v. 1.10; GATK v 4.1.2; ShatterSeek v0.4; ChainFinder v1.0.1; fastSTRUCTURE v1.0; Pophelper v2.2.7; ActiveDriverWGS v1.0.1; GISTIC v2.0.23; CNVkit v0.9.6; Manta v1.6.0; GRIDSS v2.8.3; fishHook v0.1; Bionano Access 1.5.2; ConsensusClusterPlus v1.50.0; ActivePathways v1.0.2; TitanCNA snakemake workflow v1.17.1.

For manuscripts utilizing custom algorithms or software that are central to the research but not yet described in published literature, software must be made available to editors and reviewers. We strongly encourage code deposition in a community repository (e.g. GitHub). See the Nature Portfolio guidelines for submitting code & software for further information.

Data

Policy information about availability of data

All manuscripts must include a data availability statement. This statement should provide the following information, where applicable:

- Accession codes, unique identifiers, or web links for publicly available datasets
- A description of any restrictions on data availability
- For clinical datasets or third party data, please ensure that the statement adheres to our policy

DNA sequence data have been deposited in the European Genome-Phenome Archive (EGA; https://ega-archive.org) under overarching accession EGAS00001006425 and including the Southern African Prostate Cancer Study (SAPCS) Dataset EGAD00001009067 and Garvan/St Vincent's Prostate Cancer Database EGAD00001009066. Academic researchers meeting the Data Access Policy criteria, may apply for data access via the respective Data Access Committees. CPGEA data are available via http://www.cpgea.com. PCAWG data are available at ICGC Data Portal (https://dcc.icgc.org/releases/PCAWG).

Field-specific reporting

Please select the one below that is the best fit for your research. If you are not sure, read the appropriate sections before making your selection.

☒ Life sciences ☐ Behavioural & social sciences ☐ Ecological, evolutionary & environmental sciences

For a reference copy of the document with all sections, see nature.com/documents/nr-reporting-summary-flat.pdf

Life sciences study design

All studies must disclose on these points even when the disclosure is negative.

Sample size

Sample sizes were determined in order to obtain nearly 200 tumour-blood pairs, based on biospecimen availability, with a focus on samples of underrepresented populations. All primary tumours and matched-blood tissue from 190 specimens were used to generate sequencing data in this study. We considered this sample size would be sufficient because our significant comparison of tumour genome profiling between Europeans (n=9) and Africans (n=6) has been previously published in a peer-reviewed journal. For comparisons, 93 CPGEA donors were included due to high-risk prostate cancer with most treatment-naive and 628 PCAWG donors were chosen based on different primary ancestries. Additional 256 prostate cancer patients from PCAWG were compared with most treatment-naive.

Data exclusions

After quality assurance, data from 8 tumour-blood pairs were excluded as unusable. Reasons for data exclusion included evidence of cross-contamination and duplication. Hypermutated tumours (30 mutations/Mb) were removed in mutational recurrence analysis of small mutations and cancer evolution analysis , following ActiveDriverWGS and PhyloWGS software user manuals.

Replication

The accuracy of SV breakpoint inference was assessed by applying two different algorithms and selecting only calls detected by both. Integrative clustering analysis was re-assessed using independent clustering of each dataset, with subsequent results mostly recapitulating the subtypes found by the integrative analysis.

Randomization

N/A - This exploratory study of genome profiling tumours in underrepresented populations did not contain a randomisation step due to biospecimen scarcity.

Blinding

N/A - This exploratory study within underrepresented populations did not contain a blinded data collection due to the focus on those populations in this study. Sequencing and early steps in data analysis were partially blinded using a pool of all samples of different ancestries collected.

Reporting for specific materials, systems and methods

We require information from authors about some types of materials, experimental systems and methods used in many studies. Here, indicate whether each material, system or method listed is relevant to your study. If you are not sure if a list item applies to your research, read the appropriate section before selecting a response.

Materials & experimental systems

n/a	Involved in the study
☒	☐ Antibodies
☒	☐ Eukaryotic cell lines
☒	☐ Palaeontology and archaeology
☒	☐ Animals and other organisms
☐	☒ Human research participants
☒	☐ Clinical data
☒	☐ Dual use research of concern

Methods

n/a	Involved in the study
☒	☐ ChIP-seq
☒	☐ Flow cytometry
☒	☐ MRI-based neuroimaging

Human research participants

Policy information about studies involving human research participants

Population characteristics

Patient-by-patient clinical data are provided in Supplementary Table 1. Demographically, the cohort included 53 Australians, 7 Brazilians and 123 Africans, with ages ranging from 45-99 years old (median 65.5 yo). Having performed genetic ancestry analysis, the cohort consisted of 113 Africans, 61 Europeans and 9 Admixed mostly between African and European. Preoperative PSA levels ranged from 3.5 to 4,847 ng/ml (median 22.9 ng/ml). ISUP Grade Groups were distributed as follows: 0-2: 29 (16.6%); 3: 11 (6.3%); 4: 52 (29.7%); and 5: 83 (47.4%). All patients are male.

Recruitment

After obtained the consent of patients, 183 patients from Australia (n=53), Brazil (n=7) and South Africa (n=123) and presenting mostly with clinicopathologically confirmed prostate cancer had their tumour and blood samples collected. All except one Australian patient (PID 15178) treated with one-month-long Ozurdex therapy were treatment naïve at time of sampling. Three patients were unconfirmed for the cancer and confirmed for benign prostate hyperplasia (BPH). All men from the Southern African Prostate Cancer Study (SAPCS) were recruited at the time of diagnosis, and therefore tumour tissue was derived from biopsy core, while age and PSA levels were recorded at the time of diagnosis. Australian and Brazilian subjects were recruited at the time of radical prostatectomy. Additional selection criteria included: availability of fresh-frozen tissue and matched blood, self-reported ethnicity and country of origin, as well as availability of clinical and pathological data.

Results in this study would not represent all underrepresented populations in Africa and South America as only South Africa and Brazil were studied.

Ethics oversight	All samples were obtained with written informed consent, as per study approval granted from the St. Vincent's Human Research Ethics Committee in Australia (HREC), SVH/12/231, the Grupo de Pesquisa e Pós-Graduação (GPPG) Scientific Committee and Research Ethical Commission (IRB) approval number 20160539 in Brazil or the University of Pretoria Faculty of Health Sciences Research Ethics Committee (with US Federal wide assurance FWA00002567 and IRB00002235 IORG0001762) approval number 43/2010 in South Africa. Samples were shipped to the Garvan Institute of Medical Research in accordance with institutional Material Transfer Agreements (MTAs), as well as additional Republic of South Africa Department of Health Export Permit (National Health Act 2003, J1/2/4/2 No 1/12). Whole genome sequencing and analysis were performed in accordance with approval granted by St. Vincent's Hospital HREC SVH/15/227 and governance review authorisation granted for human research at the Garvan Institute of Medical Research GHRP1522.

Note that full information on the approval of the study protocol must also be provided in the manuscript.
